# Successful management of cutaneous paralagenidiosis in a dog treated with mefenoxam, minocycline, prednisone, and hyperbaric oxygen therapy

**DOI:** 10.1016/j.mmcr.2020.07.003

**Published:** 2020-07-28

**Authors:** Amelia G. White, Kimberly Smart, Terri Hathcock, D. Michael Tillson, Anil Poudel, Patricia Rynders, Chengming Wang

**Affiliations:** a Auburn University College of Veterinary Medicine, Department of Clinical Sciences, 1220 Wire Road, Auburn, AL, 36849, USA; b BluePearl Vet Hospital, 13160 Magisterial Drive, Louisville, KY, 40223, USA; c Auburn University College of Veterinary Medicine, Department of Pathobiology, 264 Greene Hall, Auburn, AL, 36849, USA; d Auburn University College of Veterinary Medicine, Department of the University Veterinarian, 311 Greene Hall, Auburn, AL, 36849, USA

**Keywords:** *Paralagenidium* sp.Oomyces, Fungal dermatitis, *Pythium* sp., Mefenoxam, Minocycline

## Abstract

Cutaneous oomycotic infections are a rare dermatological disease primarily affecting horses and dogs. Response to medical management with antifungal therapies is poor because these organisms are not true fungi. Complete cure is unlikely if the infected tissue is unable to be completely surgically excised. This is a case report of successfully-managed cutaneous paralagenidiosis infection of the perianal tissue in an 11-month-old male intact Labrador retriever utilizing hyperbaric oxygen therapy, corticosteroids, minocycline, mefenoxam, and surgery.

## 1 Introduction

Infections caused by members of the class Oomycetes represent a group of emerging diseases in both human and veterinary medicine. Organisms within this group commonly are referred to as water molds and many are economically important plant, fish, and crustacean pathogens [1]. Oomycetes are filamentous, fungal-like organisms that are found in warm, aquatic environments and are more closely related to algae than fungi possessing cellulose and β-glucan rather than chitin or ergosterol as the primary component of the cell wall or membrane, respectively. For some time, *Pythium insidiosum* was the only Oomycota known to be a mammalian pathogen. This changed in 2003 when several cases of invasive cutaneous infections by an organisms resembling *Pythium* sp. were reported [2]. Subsequent studies determined these infections to be due to the genera *Lagenidium* sp. and *Paralagenidium* sp [3]. Similar to *Pythium* sp., infection is thought to occur through penetration of non-intact epithelial membranes by motile, biflagellated zoospores released into slow-moving or stagnant fresh water. In the United States of America, oomycotic infections most commonly occur in the Gulf Coast states, but have been reported in several other states across the southeastern, midwestern, and western regions of the country including Arizona [4].

Clinical disease due to *Lagenidium* sp. and *Paralagenidium* sp. in dogs has many features similar to cutaneous pythiosis [2,4]. Grossly, the lesions appear as firm dermal or subcutaneous nodules or as ulcerated, thickened, edematous areas with necrosis and draining tracts. Histopathologic features include pyogranulomatous inflammation with or without eosinophils and the presence of broad, irregularly branching, poorly septate hyphae with nonparallel walls [4]. Typically, *Lagenidium* sp. and *Paralagenidium* sp. have a larger hyphal diameter than *Pythium* sp., and, in contrast to *Pythium* sp., are usually visible on hematoxylin and eosin (H&E) stained sections [2,4]. The gold standard of therapy for these infections typically involves surgical excision with concurrent systemic antifungal therapy, most commonly itraconazole and terbinafine. In general, the prognosis ranges from poor to grave for oomycotic infections.

The case presented here is an example of success medical management of cutaneous paralagenidiosis in the perianal region of a dog using non-traditional antifungal therapies.

## 2 Case

### 2.1 Case presentation

An 11-month-old male intact Labrador retriever presented to our tertiary referral center (Day 0) for routine castration surgery and evaluation of chronic perianal dermatitis of four weeks duration. The dog was part of a working colony with exposure to many different environments including fields, ponds, and woods in the southeastern region of the United States of America.

**Fig. 1:**
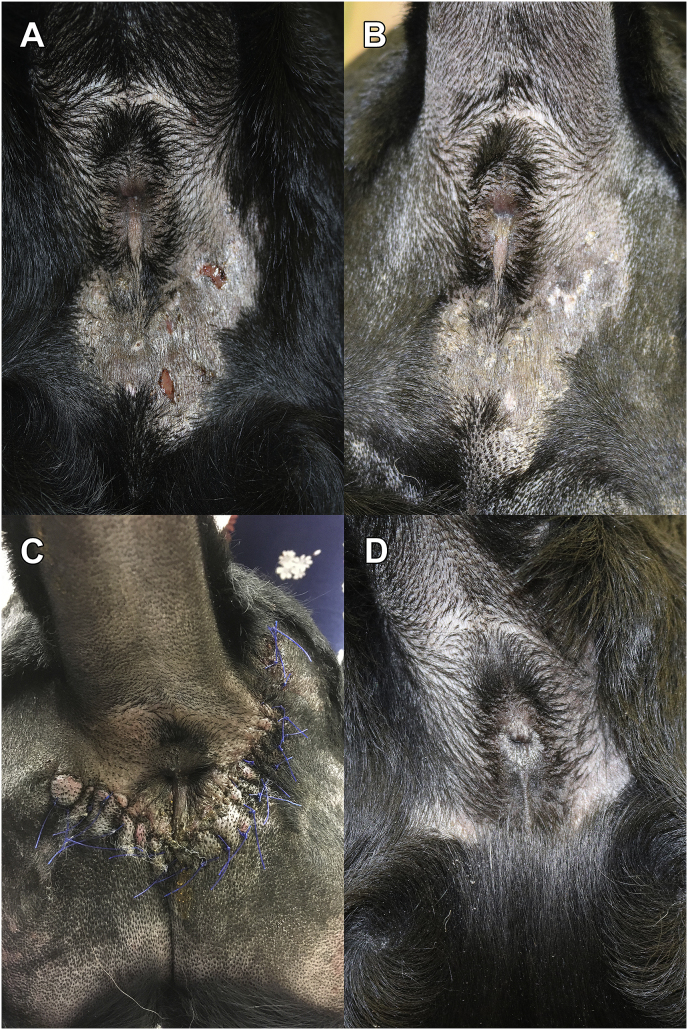
Clinical lesion progression over time. (A) The perianal area contained a well-demarcated, asymmetrical, erythematous area of lichenification with papules, crusts, punctate ulcerations and fistulae. The affected area extended clockwise around the anus from the 12 o'clock to 7 o'clock positions. The affected area to the right of the anus measured 3.2 cm (length, L) × 3 cm (width, W) × 1 cm (depth, D). The affected area ventral to the anus measured 5.5 cm (L) × 5 cm (W) × 1 cm (D). (B) Day +77 after completion of nine “dives” over a five week period, the tissue was grossly normal; however, a depigmented well-defined area remained at the site of previous inflammation. (C) Day +84 demonstrating the incomplete surgical resection of the affected perianal tissue. (D) Day +170 the lesion was completely healed with a remaining scar and all treatments were discontinued.

The perianal area contained a well-demarcated, asymmetrical, erythematous area of lichenification with papules, crusts, punctate ulcerations and fistulae. The affected area extended clockwise around the anus from the 12 o'clock to 7 o'clock positions (Fig. 1A).

### 2.2 Diagnostic evaluations

Superficial and deep skin scrapings revealed no evidence of ectoparasites at ×100 total magnification. Cytological examination revealed neutrophils and macrophages with intracellular and extracellular cocci bacteria at ×1000 total magnification in oil immersion indicative of pyogranulomatous inflammation with secondary bacterial dermatitis.

During surgical castration on Day 0, four 6 mm full-thickness cutaneous punch biopsies were performed on the affected perianal lesion. Tissue samples were submitted for histopathological evaluation, aerobic culture and susceptibility, anaerobic culture, and fungal culture.

**Fig. 2:**
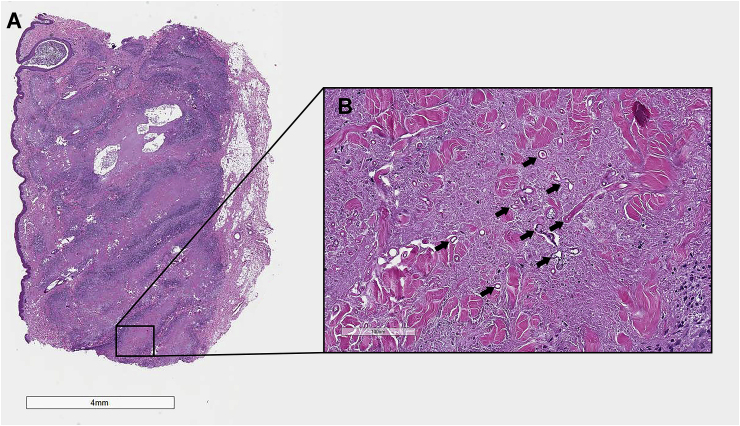
Histopathology of cutaneous lesion, H&E. (A) Photomicrograph of lesion histopathology (H&E − Hematoxylin and eosin,×10 magnification). Histopathological examination of the skin biopsies revealed chronic, severe pyogranulomatous and necrotizing nodular dermatitis and panniculitis with intralesional fungal hyphae measuring 6–14 μm in width with non-parallel walls, irregular non-dichotomous branching, frequent septation, and occasional bulbous terminal dilatations. (B) Photomicrograph of histopathology (H&E, ×200 magnification). Eosinophilic-staining hyphae (black arrows) within pyogranulomatous dermatitis are easily visible.

**Fig. 3:**
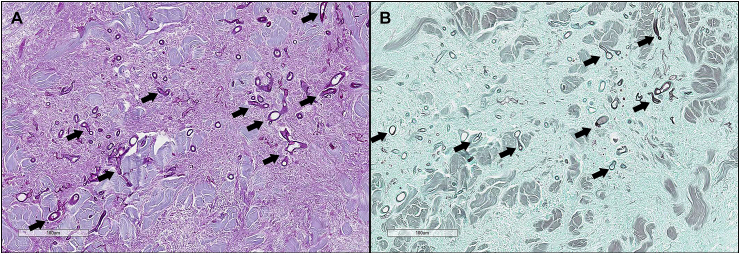
Histopathology of cutaneous lesion, PAS and GMS. (A) Photomicrograph of lesion histopathology (PAS – Periodic acid-Schiff, ×200 magnification). Eosinophilic-staining hyphae within pyogranulomatous dermatitis are easily visible. Hyphae measure 6–14 μm in width with non-parallel walls, irregular non-dichotomous branching, frequent septation, and occasional bulbous terminal dilatations (black arrows). (B) Photomicrograph of histopathology, special stain (GMS – Grocott methenamine silver, ×200 magnification). Dark-staining hyphae within pyogranulomatous dermatitis are easily visible (black arrows).

Histopathological examination of the skin biopsies revealed chronic, severe pyogranulomatous and necrotizing nodular dermatitis and panniculitis with intralesional fungal hyphae. The dermis and subcutis were markedly expanded by coalescing granulomas characterized by large foci of necrosis and cavitation bordered by degenerate neutrophils, circumferential bands of epithelioid and fewer multinucleated giant cell macrophages, and peripheral infiltrates of lymphocytes and plasma cells. Within areas of necrosis were moderate numbers of hyphae measuring 6–14 μm in width with non-parallel walls, irregular non-dichotomous branching, frequent septation, and occasional bulbous terminal dilatations. Hyphae were highlighted by the Periodic acid-Schiff (PAS) reaction and Grocott-Gomori's methenamine silver (GMS) stain, and did not have any immunoreactivity for *Pythium* sp. antigen with immunohistochemistry (Fig. 2, Fig. 3) [5].

Aerobic culture and antimicrobial susceptibility testing revealed moderate growth of methicillin-susceptible *Staphylococcus pseudintermedius*. The anaerobic culture was negative. Potassium hydroxide digests of tissue microscopically demonstrated tissue invasion with irregular hyphae producing little to no branching. The fungal culture was positive for pale, submerged colonies with no distinct pattern of growth consistent with *Paralagenidium* sp. The whole genome of this isolate (designated *Paralagenidium karlingii* strain 1391) was sequenced at Hudson Alpha Genomic Services Laboratory (Huntsville, AL, USA) using an Illumina HiSeqX platform, and has been deposited at DDBJ/ENA/GenBank under the accession PTTM00000000 (www.ncbi.nlm.nih.gov/nuccore/PTTM00000000) as described [6].

Serum was collected and submitted to the Auburn University College of Veterinary Medicine Infectious Disease Laboratory (Auburn, AL, USA) for *Pythium* serological evaluation. This enzyme-linked immunosorbent assay (ELISA) for detection of *Pythium* sp. quantifies results as percent (%) positivity with active pythiosis ranging from 40 to 100% positive while normal (uninfected) dogs range from 5 to 10% positive. Results from this dog revealed 7% positivity at 1:1000 dilution, 6% positivity at 1:2000 dilution, and 1% positivity at 1:4000 dilution consistent with a negative result.

Once a diagnosis of cutaneous paralagenidiosis was confirmed, a systemic workup was performed to assess for disease dissemination to internal organs (Day +42). Diagnostics included abdominal and thoracic radiographs, abdominal ultrasonography, complete blood cell count, biochemical profile, and urine analysis. All diagnostic results were unremarkable with no evidence of disease dissemination.

### 2.3 Differential diagnoses

Considering the signalment and environmental history, the primary differential diagnosis was an opportunistic infectious dermatitis. Other less likely differentials included sterile nodular panniculitis, sterile pyogranuloma granuloma syndrome, perianal fistula, and neoplasia.

### 2.4 Treatment and outcome

Infection with oomycotic organisms is considered a guarded to poor prognosis despite aggressive medical and surgical therapy [[2], [3], [4]]. The location of this dog's infection caused concerns for incomplete surgical resection and a high risk of surgically-induced rectal atony, paresis, or even paralysis. Aggressive medical treatment was initiated on Day +48 in an effort to reduce the grossly diseased area prior to surgical debulking. Minocycline (10 mg/kg q12 h per os) was administered due to its *in vitro* inhibitory effects against the oomycete *Pythium* sp [7,8]. Prednisone (0.5 mg/kg/day per os) was administered on a four-week tapering schedule to alleviate pain and reduce tissue swelling [9,10]. Mefenoxam is a plant fungicide known to have *in vitro* killing properties against *Pythium* sp., and was administered to treat the oomycotic infection (8 mg/kg/day per os, Subdue Maxx® Fungicide, Syngenta®, Greensboro, North Caroline, USA) [11].

In addition to the oral therapies, hyperbaric oxygen therapy (HBOT) was used to improve oxygenation of the diseased tissue, reduce pain and swelling, decrease microbial translocation, and promote wound healing [12]. The dog completed nine 45 minute sessions of HBOT at 2 atm absolutes (ATA). One hundred per cent oxygen was flowing throughout with 15 minute control descend and ascend (“dive”) periods in each session. A total of nine “dives” were performed over a five-week period on Days +44, 49, 51, 54, 58, 62, 64, 69, and 77. After the first HBOT session (Day +44), the affected area was no longer palpably edematous. By the third HBOT session (Day +51), tissue edema, inflammation, and fistulae were completely resolved with minor scale remaining (Fig. 1B). After completion of nine “dives” over a five week period (Day +77), the tissue was grossly normal; however, a depigmented well-defined area remained at the site of previous inflammation.

Due to the radical nature of the treatment protocol, the patient was monitored for adverse effects every one to two weeks with a physical examination, complete blood count, heparinized whole blood biochemistry profile, and urine analysis. No adverse events or significant abnormalities were noted on diagnostic evaluations.

On Day +84 following completion of HBOT treatments, aggressive surgical resection was performed by a board-certified veterinary surgeon (Fig. 1C). Fresh tissue from the remaining surgical dorsal, right and left lateral, and deep surgical margins was submitted for fungal culture and histopathology. The fungal culture was positive for *Paralagenidium* sp. on the dorsal and right lateral margins indicating incomplete excision. Histopathology of the resected tissue similarly revealed fungal hyphae at the surgical margins. Treatment with minocycline and mefenoxam was continued for three months post-operatively until Day 170 (Fig. 1D). No additional HBOT sessions were performed following surgical debulking due to treatment expense. The previously described in-depth monitoring for adverse events was continued every two weeks until Day +170. Follow-up visits continued every two to three months thereafter for twelve months, and then every three to six months thereafter.

Over the course of the five-month treatment with oral therapies, the patient did not experience any gastrointestinal or hematological abnormalities. The medications appeared safe, effective, and were easy to administer in this single patient. The surgical site healed normally and hair regrowth occurred. At Day +1020 after discontinuation of all treatments, there is no evidence of disease recurrence.

## 3 Discussion

This is the first case report of successful resolution of cutaneous paralagenidiosis in a dog treated with a combination of non-traditional antifungal therapies including mefenoxam, HBOT, and surgery. The treatment was effective and no immediate or delayed adverse side effects were noted in this dog.

Traditional therapies used in the treatment of oomycotic infections include aggressive surgical resection in combination with multimodal antifungal therapy using terbinafine, azoles, and/or amphotericin B. While this traditional treatment protocol has shown some success to reduce fungal growth, these drugs have poor efficacy rates for disease cure. Most of these drugs target components of the fungal cell wall or membrane leading to organism death, but these molecules are not a major component in oomycotic cell membranes rendering these drugs poorly effective. If resolution is not achieved, oomycotic infections have a high rate of mortality due to systemic dissemination of infection [4]. Immunotherapy utilizing *Pythium* sp. antigens is a treatment modality that has shown success in horses with pythiosis, but the results are not as promising in dogs and there are no reports that this therapy is successful for other oomycotic infections including *Lagenidium* sp. and *Paralagenidium* sp [13,14]. The lack of success with currently available treatment options supports the need for developing new efficacious and safe non-traditional options.

The genus *Paralagenidium* is the most recently identified organism in the class Oomycetes, and little published data exists to support successful treatment efforts with paralagenidiosis cases [3]. It is known, however, that its clinical disease carries a better prognosis than other oomycotic organisms because progression is much slower with lower risk for systemic dissemination. Aggressive surgical approach can be curative for paralagenidiosis if complete resection with wide margins is achieved followed by prolonged antifungal therapy [15]. If the infection is in an area where complete resection is not obtainable, then antifungal therapy alone is not an effective treatment approach.

This case presented the challenge of infection in a body location not amenable to aggressive surgery without the risk for significant post-operative complications. The best approach was to use medical therapy to reduce the size of the affected tissue prior to surgical resection. Attention was focused on identifying non-traditional methods of therapy because of the known poor response to traditional antifungal medications for oomycotic infections. When researching options, several *in vitro* studies showed efficacy of antibiotic, antiprotozoal, and plant fungicidal agents as potentially promising future treatments. In a study by Brown et al., the agricultural fungicide mefenoxam at 1 μm/mL achieved 90% inhibition of growth of *Pythium insidiosum* and *Lagenidium* sp. Mefenoxam use was cited in a single case report of successful medical treatment of gastrointestinal pythiosis in a dog [11]. This report utilized mefenoxam at four mg/kg per os every 12 h in combination with terbinafine for 18 months with no reported adverse side effects. These two sources sparked our interest in utilizing mefenoxam as a non-traditional therapy agent for this case of cutaneous/subcutaneous paralagenidiosis.

Mefenoxam is an agricultural fungicide used to prevent and treat pathogenic fungal and oomycotic pathogens of crops through inhibition of ribosomal ribonucleic acid (RNA) polymerases [16]. Preliminary safety studies presented to the United States Environmental Protection Agency (EPA) reported that a dose of 8 mg/kg/day per os resulted in no-observable-effect-level (NOEL) in a six month feeding study performed in dogs [17]. While the pharmacokinetics and pharmacodynamics of this product in the dog is unknown, it has been extrapolated that this dose may achieve plasma drug concentrations greater than 1 μg/mL which is the minimum required concentration for *in vitro* killing of *Pythium* sp. and *Lagenidium* sp [11]. Similar to other reported medical uses of mefenoxam in dogs, mefenoxam was well-tolerated in the dog in this case report and no adverse acute or chronic clinical, gastrointestinal, hematological, or biochemical effects were noted for over two years after discontinuation of the therapy [11].

Minocycline is a tetracycline antibiotic that inhibits protein synthesis through targeting the 30S ribosomal subunit. Several antibiotics targeting protein synthesis have demonstrated *in vitro* effectiveness against *Pythium* sp.; however, no studies have evaluated activity against *Lagenidium* sp. and *Paralagenidium* sp [7,8,18]. There are reports of successful minocycline use *in vivo* for people with ocular fungal keratitis but no published reports of its use for oomycotic infections in veterinary medicine [19]. Loreto et al. reported that the minimum inhibitory concentration (MIC)90 for *Pythium* sp. *in vitro* is 2 μg/mL [18]. Minocycline administered orally to fasted dogs at the mean dose 4.99 mg/kg produces maximum blood concentrations of 2.27 μg/mL [20]. This data was extrapolated to determine that a dose of 10 mg/kg q 12 h per os would achieve a blood concentration above 4 μg/mL.

Hyperbaric oxygen therapy (HBOT) is an emerging treatment modality in veterinary medicine with many promising benefits. Utilizing a controlled pressurized system, oxygen saturation occurs within various tissues inside the body and is postulated to accelerate healing and enhance drug penetration at sites of inflammation [12]. It commonly is recommended as adjunctive therapy for severe wound management, vasculitis, deep tissue infections, snake envenomation, and a variety of neuropathies in veterinary medicine. Having easy access to HBOT within our tertiary facility and knowing the proposed positive effects on wound management, it was decided to incorporate this into our multi-modal protocol. A noticeable reduction in tissue edema and erythema was noted within the first three days of HBOT therapy prior to the initiation of oral medical therapies suggesting that this treatment modality successfully reduced inflammation in this dog.

It is not known what role each aspect of therapy may have played in the success of this dog's treatment success or if this multimodal approach will yield similar results in other dogs. It also is unknown what long-term health risks could be associated with the off-label use of mefenoxam in this case or in other dogs. Further safety evaluation and pharmacokinetic/pharmacodynamic studies are needed to assure its routine use for the treatment of oomycotic infections in dogs. Use of mefenoxam was reported as a safe and efficacious treatment in a single case report of a dog diagnosed with gastrointestinal pythiosis, and appeared safe and efficacious in this dog as well [11]. The successful management of incompletely resected cutaneous paralagenidiosis in this case supports the discussion and future investigation into emerging treatment options given the poor prognosis associated with this infectious disease.

## Ethical form

This case report was self-funded. This research did not receive any specific grant from funding agencies in the public, commercial, or not-for-profit sectors.

Written and signed consent to publish the case report was provided by the dog owner.

## Declaration of competing interest

There are no conflicts of interests.
